# Dendritic cells in atherosclerotic inflammation: the complexity of functions and the peculiarities of pathophysiological effects

**DOI:** 10.3389/fphys.2014.00196

**Published:** 2014-05-27

**Authors:** Dimitry A. Chistiakov, Igor A. Sobenin, Alexander N. Orekhov, Yuri V. Bobryshev

**Affiliations:** ^1^Department of Medical Nanobiotechnology, Pirogov Russian State Medical UniversityMoscow, Russia; ^2^Skolkovo Innovative Center, Institute for Atherosclerosis ResearchMoscow, Russia; ^3^Institute of General Pathology and Pathophysiology, Russian Academy of SciencesMoscow, Russia; ^4^Laboratory of Medical Genetics, Russian Cardiology Research and Production ComplexMoscow, Russia; ^5^Faculty of Medicine, School of Medical Sciences, University of New South Wales, Kensington, SydneyNSW, Australia

**Keywords:** atherosclerosis, atherogenesis, inflammation, immune reactions, dendritic cells, arteries

## Abstract

Atherosclerosis is considered as a chronic disease of arterial wall, with a strong contribution of inflammation. Dendritic cells (DCs) play a crucial role in the initiation of proatherogenic inflammatory response. Mature DCs present self-antigens thereby supporting differentiation of naïve T cells to effector cells that further propagate atherosclerotic inflammation. Regulatory T cells (Tregs) can suppress proinflammatory function of mature DCs. In contrast, immature DCs are able to induce Tregs and prevent differentiation of naïve T cells to proinflammatory effector T cells by initiating apoptosis and anergy in naïve T cells. Indeed, immature DCs showed tolerogenic and anti-inflammatory properties. Thus, DCs play a double role in atherosclerosis: mature DCs are proatherogenic while immature DCs appear to be anti-atherogenic. Tolerogenic and anti-inflammatory capacity of immature DCs can be therefore utilized for the development of new immunotherapeutic strategies against atherosclerosis.

## Introduction

**Figure 1:**
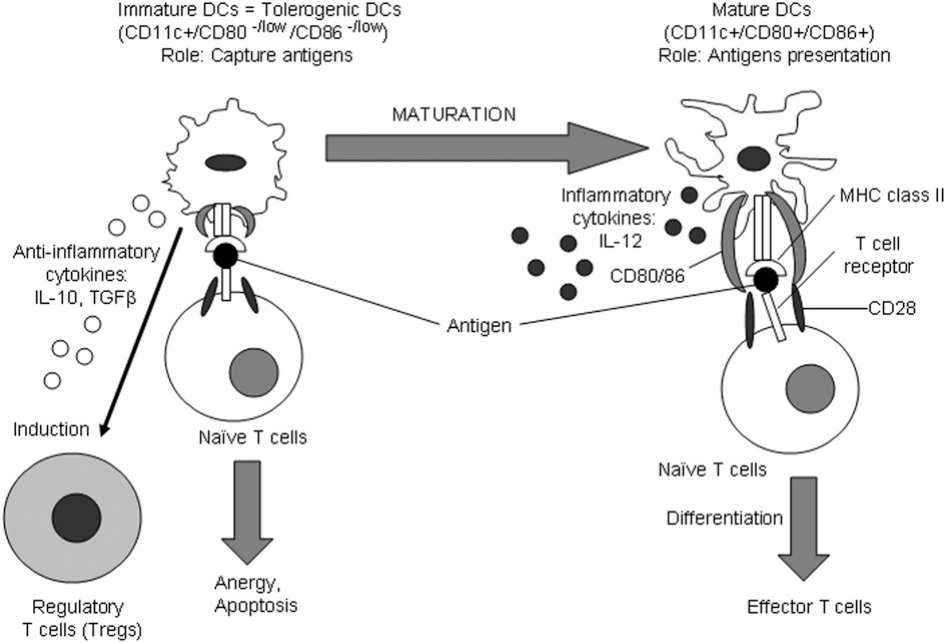
**Immature and mature DCs**. Immature DCs arise from DC precursors that circulate in the blood. From the blood, DC precursors may reach the target tissue where they transform into immature DCs. The major function of immature DCs is the capture of antigens. Immature DCs capturing antigens may then migrate into lymphoid tissues such as the spleen and lymph nodes where they further differentiate becoming mature DCs. Mature DCs are capable to efficiently present captured antigens to naïve T cells. DC maturation is characterized by the up-regulation of expression of molecules responsible for antigen presentation such as MHC class II and CD80/CD86. After antigen recognition, naïve T cells differentiate into effector T cells. Differentiation of naïve T cells to effector cells may occur upon cell-cell contacts between a DC and a naïve T cell and *via* proinflammatory cytokine IL-12 secreted by mature DCs. Immature DCs lacking sufficient expression of antigen-presenting molecules cause anergy and apoptosis of naïve T cells. Immature DCs possess tolerogenic properties by inducing Tregs through cell-to-cell contact with naïve T cells and through secreting anti-inflammatory cytokines such as IL-10 and TGF-β. Since immature DCs are capable to induce Tregs and inhibit the inflammatory reaction in the atherosclerotic plaque, the development of strategies for the induction of tolerogenic DCs is of great therapeutic promise.

Dendritic cells (DCs) were first described by Steinman and Cohn (1973). DCs are a heterogeneous group of professional antigen-presenting cells (APCs). DCs differentiate from precursors circulating in the bloodstream (Sorg et al., 1999). The precursors can be delivered by blood to the target non-lymphoid organ or tissue, in which they become immature DCs. Immature DCs express on their surface integrin alpha X (CD11c) (Sorg et al., 1999). However, costimulatory molecules CD80/CD86 essential for T cell activation are not or expressed or produced at very low levels (Cocks et al., 1995) (Figure 1). Indeed, immature DCs can capture antigens but are not able to stimulate naïve T cells. From the target tissue, immature DCs in turn migrate to lymphoid organs such as spleen and lymph nodes in which they differentiate into mature DCs. The maturation is accompanied by enhanced expression of costimulatory molecules CD80/86 and CD40, antigen-presenting molecules (MHC class I and II), and adhesion molecules (CD11a, CD50, CD54, CD86). Mature DCs can contact and present antigens to naïve T cells, which in turn differentiate into effector T cells such T helper 1 (Th1) or 2 (Th2) cells. DCs also secrete interleukin (IL)-12, a proinflammatory cytokine that directs differentiation of naïve T cells to effector T cells (Kubin et al., 1994). Some immature DCs exhibit tolerogenic properties through the induction of regulatory T cells (Tregs) and suppression of naïve T cell activation (Steinman et al., 2003).

Atherosclerosis is a chronic inflammatory disease, with a pathogenic immune response driven by T lymphocytes (Hansson and Hermansson, 2011). Due to their critical role in effector T cell differentiation from naïve T cells, it is not surprisingly that DCs are found to be the key players in the proinflammatory response at the atherosclerotic plaque. The discovery of the presence of DCs in the intima of normal arteries and atherosclerotic lesions led to a suggestion that DCs might play an important role in the development of atherosclerotic lesions (Bobryshev and Lord, 1995a, b). An entire network of HLA-DR-expressing cells was eventually found to exist in the intimal space of normal human aortas (Bobryshev et al., 2012), suggesting their potential role in the regulation of vascular homeostasis. The architectonics of this network may be different in various aortic segments thereby predicting putative atherosclerosis-prone and atherosclerosis-resistant regions of the visually normal aorta (Bobryshev and Lord, 1995a).

The involvement of DCs in atherogenesis was then proven by experimental findings on two rodent atherosclerosis models such as apolipoprotein (apo) E-null and LDL receptor (LDLR)-null mice (Bobryshev et al., 1999; Paulson et al., 2010). Transfer of oxidized low density lipoprotein (oxLDL)-reactive T cells to ApoE-null severe combined immunodeficiency syndrome (Scid) mice are more effective in plaque presentation compared to the transport of non-specific T cells to an antigen derived from a lesion (Zhou et al., 2006), thereby suggesting on the involvement of those cells in presenting antigens in disease progression. DCs were observed in atherosclerotic lesions of apoE- (Bobryshev et al., 1999, 2001) and LDLR-null mice (Paulson et al., 2010), and these cells were not present in the plaques just accidentally but accumulated and contributed to the intraplaque inflammation and progression of the coronary atheroma (Ludewig et al., 2000; Hjerpe et al., 2010).

Since the identification of DCs in the arterial wall (Bobryshev and Lord, 1995a, b), the functional significance of this cell type has been intensely studied and various issues of the involvement of DCs in atherogenesis have been discussed in a number of reviews (Bobryshev, 2000, 2005, 2010; Cybulsky and Jongstra-Bilen, 2010; Niessner and Weyand, 2010; Koltsova and Ley, 2011; Manthey and Zernecke, 2011; Van Vré et al., 2011; Butcher and Galkina, 2012; Feig and Feig, 2012; Takeda et al., 2012; Alberts-Grill et al., 2013; Cartland and Jessup, 2013; Grassia et al., 2013; Koltsova et al., 2013; Subramanian and Tabas, 2014). It is important to note here that functions of DCs in human arteries are still practically unknown and that the accumulated information about functions of DCs in atherosclerosis is obtained in experimental studies. However, in contrast to the intima of human arteries which contains the nets of DCs (Bobryshev and Lord, 1995a; Bobryshev, 2000), the intima of large arteries in animal models of atherosclerosis consists of the endothelium that is separated from the internal elastic membrane by just a narrow layer of free-of-cell matrix. In humans, the activation of resident vascular DCs occurs in the very earlier stage of atherosclerosis (Bobryshev and Lord, 1995a; Bobryshev, 2000), whereas the accumulation of DCs in the arterial intima in animal models of atherosclerosis occurs as a result of the penetration of DCs or DC precursors from the blood stream, parallel with the development of atherosclerotic lesions (Bobryshev et al., 1999, 2001). Likewise, little is known about the peculiarities of functions of immature DCs *vs* mature DCs in human atherosclerosis (Bobryshev, 2010).

Accumulated evidence obtained in experimental studies indicates that, depending on the maturation stage, DCs play a double role in atherogenesis: mature DCs display proatherogenic features whereas immature DCs seem to be anti-atherogenic. In this review, we highlight a double role of DCs in atherogenesis. Obviously, further studies are needed in order to translate knowledge obtained in experiments to human atherogenesis.

## DC subsets and their role in atherosclerosis

There are several subsets of DCs can be distinguished including conventional (myeloid) DCs (cDCs), plasmacytoid DCs (pDCs), and inflammatory DCs (Shortman and Naik, 2007). Inflammatory DCs cannot be found in the steady state but emerge after inflammatory stimuli. Unlike cDCs, pDCs are weak in antigen presentation (Shortman and Naik, 2007). However, pDCs are potent inducers of the interferon (IFN) type I response against viral and bacterial infections (Villadangos and Young, 2008). Choi et al. (2011) have proposed markers to discriminate DCs subsets in murine atherosclerosis but the function of different subsets remains to be elucidated. Although the presence of both subtypes of DCs is known in humans, the most reliable information about the functions of cDCs and pDCs are obtained in animal studies (Shortman and Naik, 2007; Villadangos and Young, 2008; Busch et al., 2014).

Normally, vascular DCs were proposed to contribute to maintaining tolerance to self-antigens; in proatherosclerotic conditions, activated vascular DCs may present self-antigens to T cells and promote inflammatory response in the plaque (Niessner and Weyand, 2010). Both pDCs and cDCs were found in the shoulder region of carotid artery lesions (Niessner et al., 2006). pDC were detected in human and mouse atherosclerotic lesions (Niessner et al., 2006; Niessner and Weyand, 2010; Daissormont et al., 2011). pDCs were shown to produce large amount of IFNα, a potent regulator of T cells, that might lead to the unstable plaque phenotype (Niessner et al., 2006). In the LDLR-null mice, pDC depletion, in contrast, led to T cell accumulation and promoted atherosclerotic lesion formation (Niessner et al., 2006). However, in a recent study, specific depletion of pDC in aortas and spleen of apoE-deficient mice was associated with significantly reduced atherosclerosis (Macritchie et al., 2012). In another study, the construction of self-response complexes loaded with patient's DNA and anti-microbial peptides enhanced early plaque formation in apoE-null mice (Döring et al., 2012).

pDS and IFNs isolated from the plaque are involved in the maturation of DCs and macrophages (Döring and Zernecke, 2012). Indeed, these findings suggest for a rather proatherogenic role of pDCs (Döring and Zernecke, 2012) that should be further elucidated. Apart from the existence of cDCs and pDCs, lesion DCs may also arise from the blood-derived monocytes that infiltrate the intima (Randolph et al., 1998). Several studies in mice and humans have addressed this issue (Jongstra-Bilen et al., 2006; Dopheide et al., 2012). Choi et al. (2011) observed in murine aorta new subtype of monocyte-derived DCs (CD11c^high^MHCII^high^CD11b^−^CD103^+^). According to Choi et al. (2011), DCs in murine atherosclerosis are primarily presented by two subsets: macrophage-colony stimulating factor (M-CSF)-dependent monocyte-derived DCs (CD14^+^CD11b^+^DC^−^SIGN^+^), and classical Flt3-Flt3L signaling-dependent cells (CD103^+^CD11b^−^). Although there might be a wide spectrum of DCs subsets in the intima of arteries, the functioning of DCs largely depends on the maturation stage (Butcher and Galkina, 2012).

Immature CD1a^+^S100^+^lag^+^CD31^−^CD83^−^CD86^−^ DCs were found in the normal aortic intima of young individuals (Millonig et al., 2001). HLA-DR^+^CD1a^+^S-100^+^ DCs were found in the normal human aorta, carotid arteries and in atherosclerotic plaques (Bobryshev and Lord, 1995a; Bobryshev, 2000). DCs are also located in the under-plaque media and in the adventitia around the *vasa vasorum* in the shoulder regions of the atherosclerotic lesion (Bobryshev and Lord, 1998). Mature CD83 positive DCs were observed in rupture-prone plaque regions in human carotid arteries (Yilmaz et al., 2004). These studies showed that heterogeneity of the DC population seems to increase progressively during the plaque progression.

There are several pathways by which DC numbers can be increased within atherosclerotic plaques. DCs and their precursors could infiltrate plaques migrating from the bloodstream by a chemokine- and adhesion molecule-dependent pathway (Niessner and Weyand, 2010). DCs can migrate to advanced lesions from the adventitia during *vasa vasorum*-associated neovascularization (Bobryshev and Lord, 1998). The third way is associated with monocytes that penetrate the intima at early atherosclerosis steps in order to differentiate into macrophages or DCs in response to inflammatory stimuli (Randolph et al., 1998). Therefore, inhibition of the activation and recruitment of increasing numbers of activated DCs may be important in preventing lesion formation.

## Role of DCs in induction of t-cell mediated proinflammatory response in the atherosclerotic lesion

As mentioned above, immature DCs having CD11c^+^CD80^−/low^CD86^−/low^ phenotype are tolerogenic DCs responsible for capturing antigens. These DCs might play anti-inflammatory role because they may induce apoptosis or anergy in naïve T cells responding to self-antigens (Kushwah and Hu, 2011). By capturing antigens, immature DCs differentiate into mature DCs. Mature CD11c^+^CD80^+^CD86*^+^* DCs, in contrast, may play a proinflammatory role by presenting self-antigens to naïve T cells, which then differentiate to effector T cells, and by secreting inflammatory cytokines such as IL-12. During the late stage of monocyte differentiation, oxLDL promote maturating DCs into IL-12-producing cells, which further support inflammatory reaction in the plaque (Perrin-Cocon et al., 2001). Furthermore, in LDLR^−/−^ mice, resident intimal DCs were shown to rapidly accumulate oxLDL and initiate nascent foam cell lesion formation (Paulson et al., 2010). By presenting antigens, mature DCs and macrophages induce adaptive T-cell mediated immune response (Paulson et al., 2010; Choi et al., 2011). However, there is conflicting evidence on the role of oxLDL (and hyperlipidemia) on the maturation and function of DCs (Perrin-Cocon et al., 2001, 2013; Ge et al., 2011; Nickel et al., 2012; Peluso et al., 2012). A recent study by Hermansson et al. (2011) revealed that native LDL can also be recognized by DCs.

**Figure 2:**
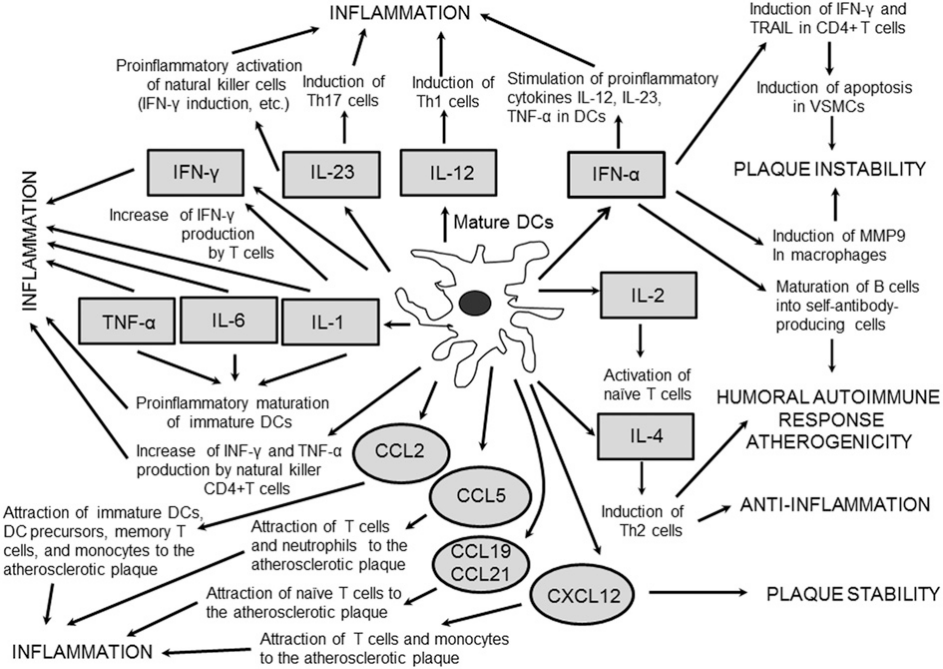
**Cytokines and chemokines produced by mature DCs in the atherosclerotic vessels**. DC-derived cytokines mostly possess proinflammatory and proatherogenic properties. Chemokines secreted by mature DCs stimulate chemotaxis of a variety of immune cells to attract them to the atherosclerotic lesion.

In shoulders of human vulnerable atherosclerotic plaques, CD83^+^ DCs were identified in the vicinity to CD40L^+^ T cells (Yilmaz et al., 2006). This population of DCs secrete CC-motif chemokines (CCL)19 and CCL21 capable to enhance recruitment of naive lymphocytes into atherosclerotic vessels (Erbel et al., 2007) (Figure 2). pDCs responded to pathogen-derived motifs and CpG-containing oligodeoxynucleotides by elevated production of IFN α that recruits naïve T cells, which then differentiate into cytotoxic CD4^+^ effector T cells capable to effectively kill vascular smooth muscle cells (VSMCs) (Niessner et al., 2006). Therefore, in the atherosclerotic lesion, pDCs are able to sense microbial motifs and accelerate activity of cytotoxic T-lymphocytes thereby providing a link between severe immune-mediated atherosclerotic complications and infections. CD4^+^ T cells derived from atherosclerotic plaques are capable to recognize oxLDL, heat shock proteins (HSP)60/65, and other antigens from pathogenic microorganisms such as *Chlamydia pneumonia*, which are supposed to be candidate self-antigens for atherogenesis (Stemme et al., 1995; Benagiano et al., 2005; Mandal et al., 2005). Stephens et al. (2008) showed the ability of a self-peptide Ep1.B derived from human apoE to induce differentiation of monocytes to DCs, thereby suggesting for a putative mechanism of self-antigen-mediated induction of inflammation at early stages of atherosclerosis. Immunochemical staining revealed the presence of *C. pneumonia* in DCs derived from atherosclerotic plaques thus supporting again a likely role of DCs as a bridge linking the pathogen-induced proinflammatory responses to the induction of atherogenesis (Bobryshev et al., 2004).

## Crosstalks between DCs and tregs in atherosclerosis

Tregs are involved in the activation, maturation, and function of DCs (Steinman and Banchereau, 2007; Chistiakov et al., 2013). By CTLA-4-dependent inhibition of CD80/CD86 expression in proatherogenic DCs, Tregs are capable to suppress their antigen-presenting activity (Onishi et al., 2008). CTLA-4 is expressed only in stimulated Tregs including forkhead box P3 (Foxp3)^+^ Tregs. Tregs. CTLA-4 interacts with B7-1 (CD80) and B7-2 (CD86) molecules broadly presented in the surface of DCs and macrophages and conducts an inhibitory signal to DCs decreasing expression of both costimulators (Bour-Jordan et al., 2011). Through binding to CD80/CD86, CTLA-4 could initiate production of indoleamine 2,3-dioxigenase (IDO) in DCs. IDO converts tryptophane to kinurenine that is a potent immunosuppressant capable to induce *de novo* formation of Tregs from naïve T cells in the local environment (Fallarino et al., 2003).

The second inhibitory pathway by which Tregs interacting with mature DCs can down-regulate antigen presentation is CD80/CD86 trogocytosis, which is a process of intercellular transfer of cell surface proteins and outer membrane fragments (Gu et al., 2012). Trogocytosis is mediated with CD28, CTLA-4, and programmed death ligand 1 (PDL-1) and involves CD80/CD86 removal from the surface of DCs therefore increasing the inhibitory capacity of Tregs. Tregs from the CTLA-4-knockdown mice failed to suppress CD80/CD86 production suggesting for a key role of CTLA-4 in the suppression of CD80/CD86 expression in antigen-presenting mature DCs (Gao et al., 2011). CTLA-4 binding to CD80/CD86 on the surface of DCs has a major role in inducing tolerance (Oderup et al., 2006). Indeed, CTLA-4 may represent a promising target for treatment of atherosclerosis by enhancing the inhibitory activity of Tregs or increasing suppression of effector T cells (Gotsman et al., 2008).

T-cell adhesion molecule lymphocyte-associated antigen 1 (LFA-1) mediates contact between an antigen-presenting DC and a Treg (Onishi et al., 2008). Tregs maintain the CD80/CD86-suppressing capacity even if potent DC-maturating stimuli are present. Thus, Tregs may effectively block proinflammatory signals from antigen-presenting DCs to naive T-lymphocytes via binding to immature DCs followed by CTLA-4/LFA-1-mediated down-regulation of CD80/CD86 production in DCs. Indeed, DCs can influence function of Tregs through their inhibition or stimulation. Proatherosclerotic CCL17^+^CD11^+^ DCs capable to down-regulate Tregs have been found (Weber et al., 2011). Also, a population of atheroprotective monocyte-derived CD11^high^MHC^high^CD11b^−^CD103^+^ DCs which are able to induce Tregs in the lesion was identified (Choi et al., 2011).

Lievens et al. (2013) have constructed an apoE-null mouse harboring a transgene whose expression causes inactivation of tumor factor growth-β receptor II (TGFβRII)-dependent signaling in CD11-positive DCs. Prevention of TGFβ RII signaling mechanism results in shifting of CD11c^+^CD8^−^ DCs toward the more proinflammatory CD11c^+^ population, which resulted in enhanced T cell activation and maturation and advanced atherosclerosis. These observations suggest for an anti-atherogenic role of TGFβ signaling that may be responsible for maintaining immunosupressory properties in DCs and preventing their differentiation toward the proinflammatory phenotype.

**Figure 3:**
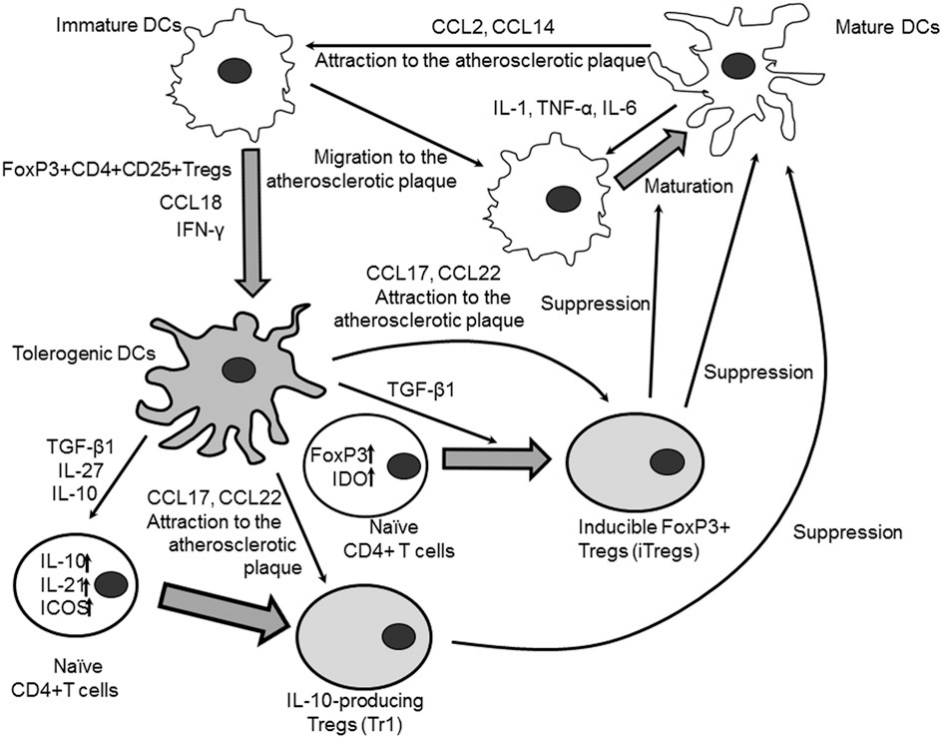
**Crosstalk between immature and mature DCs**. Mature DCs could attract immature DCs and DC precursors to the site of inflammation (i.e., to the atherosclerotic plaque) through secretion of chemokines CCL2 and CCL14. Immature DCs in turn migrate to the plaque where they are activated by the local proinflammatory microenvironment to differentiate preferentially to inflammatory mature antigen-presenting DCs. Local mature DCs could contribute to the proinflammatory DC maturation by secreting inflammatory cytokines IL-1β, IL-6, and TNF-α. Natural FoxP3^+^CD4^+^CD25^+^ T cells induce conversion of immature DCs to tolerogenic DCs. Chemokine CCL18 produced by immature DCs stimulates tolerogenicity through the induction of IL-10-mediated expression of IDO in DCs. IFN-γ is able to contribute to the formation of tolerogenic DCs by inhibiting expression of Th17-inducing osteoprotegerin and stimulating IL-27 production. IL-27 suppresses production of Th17-polarizing cytokines IL-1β, IL-6, and IL-23 from DCs and activates expression of IL-10, IL-21, and ICOS in naïve CD4^+^ T cells that drives induction of IL-10 producing Tregs (Tr1). IL-10 produced by tolerogenic DCs induces Tr1 cells through the ILT2/ILT4-mediated signaling mechanism. TGF-β 1 secreted by tolerogenic DCs could induce expression of FoxP3 and IDO in CD4^+^CD25^−^ naive T cells that promotes their conversion into inducible FoxP3^+^ Tregs (iTregs). Chemokines CCL17 and CCL22 secreted by tolerogenic CDs attract Tregs to the atherosclerotic lesion where Tregs could suppress immunomodulatory properties of proinflammatory antigen-presenting CDs and prevent differentiation of immature CDs to inflammatory subsets of mature DCs.

Most tolerogenic DCs are immature and have CD86^−^CD80^−^CD40^−^MHCII^−^ phenotype (Maldonado and von Andrian, 2010). These cells can induce Tregs by direct interaction or through production of cytokines TGF-β and IL-10. IL-10 mediates differentiation of peripheral T-cells to Tregs. Tolerogenic DCs producing IL-10 are implicated in the induction of IL-10-producing type 1 regulatory T cells (Tr1) (Figure 3). Tregs and DCs interact to each other *via* CCL17 and CCL22, and their receptors, chemokine receptors (CCR) 4 and 8 Iellem et al., 2001; Weber et al., 2011). These receptors are produced on the surface of Tregs and may bind DCs-secreted CCL17 and CCl22. CCL22 induction on tolerogenic CDs results in the recruitment of Tregs in the site of inflammation including the atherosclerotic lesion. The CCR4-deficient mice showed significantly decreased expression of IDO in DCs from mesenteric lymph nodes (Onodera et al., 2009). This finding could suggest for a role of IDO in the regulation of activating effect of CCL22 and CCR4 on Tregs induction. Overall, these observations show that the immunoregulating enzyme IDO has a crucial role in regulating reciprocal DCs-Tregs contacts mediated by B7/CTLA-4 and CCL22/CCR4 in atherogenesis. Therefore, inducing tolerogenic DCs may be important for enhancing protective effects of Trgs against atherosclerosis.

## Progress toward the clinic

In addition to the above suggestion that the development of strategies for the induction of tolerogenic DCs may be of great therapeutic promise, it is important to noting here that the accumulated evidence has showed that, indeed, DCs might be used for anti-atherosclerosis immunotherapy. The support for this view has come from a number of studies performed on mouse models of atherosclerosis in which the function of DCs was manipulated (Zhou et al., 2006; Hjerpe et al., 2010; Daissormont et al., 2011; de Jager and Kuiper, 2011; Döring et al., 2012; Macritchie et al., 2012). Currently, there is no sufficient evidence to state that cDCs are proatherogenic and that pDCs are atheroprotective. Nevertheless, based on the assumption that cDCs action is rather proatherogenic whereas pDCs might be atheroprotective, the inhibition of cDCs can induce atheroprotective immune reactions (Bobryshev, 2010).

DCs are thought nowadays as a valuable instrument for atherosclerosis immunotherapy (Van Vré et al., 2011; Takeda et al., 2012; Grassia et al., 2013; Van Brussel et al., 2013, 2014). DCs could also induce tolerance against antigens that are innate to the body (Steinman et al., 2003; Palucka and Banchereau, 2012). DCs can be used as natural adjuvants for the induction of antigen-specific T-cell responses (Caminschi et al., 2012; Palucka and Banchereau, 2012; Yamanaka and Kajiwara, 2012). Anti-atherosclerotic immunotherapy with the utilization of DCs may be the same or somewhat different from those approaches that are currently used for cancer immunotherapy (Caminschi et al., 2012; Palucka and Banchereau, 2012; Yamanaka and Kajiwara, 2012). It is essential to stress that remarkable results have been achieved in treatment of cancer patients when DCs loaded with an antigen were used as vaccines for improving the host anti-cancer immune response (Palucka and Banchereau, 2012; Yamanaka and Kajiwara, 2012). One of approaches involves an *ex vivo* treatment of DCs with an appropriate antigen, followed by further return of the antigen-treated DCs (so called “pulsed” DCs) back to the patient' blood. A similar approach can be evaluated for the use in atherosclerosis immunotherapy (Bobryshev, 2001).

One of the main challenges for developing of effective vaccines for atherosclerosis relates to the selection of a specific antigen to target. Several strategies have been offered; So far, vaccination strategies have been based on targeting of lipid antigens and inflammation-derived antigens (de Jager and Kuiper, 2011). Amongst the used approaches, immunization of hypercholesterolemic animals with oxLDL or specific epitopes of ApoB100 has been reported to inhibit atherosclerosis (Nilsson et al., 2007; van Leeuwen et al., 2009). Perhaps, DCs pulsed with autologous modified LDL or immunogenic components of autologous modified LDL could be used as well for immunization; this would allow avoiding side effects of direct vaccination with oxLDL (Bobryshev, 2010). Apart from pulsation with modified LDL, DCs can be also treated *ex vivo* through culturing with a total extract of the “own” atherosclerotic lesion, for instance, from subjects who underwent carotid enadarterectomy or other vascular interventions (Bobryshev, 2001). An obvious advantage of such approach, in which a patient is vaccinated with its “own” DCs pulsed by patient's “own” antigens is the specificity: the event of pulsation of DCs would mimic the processes that occur in plaques of the patient. This approach can be considered as an example of “personalized” medicine (Hamburg and Collins, 2010).

Although the idea to use of DCs for vaccination in atherosclerosis is widely discussed (de Jager and Kuiper, 2011; Van Vré et al., 2011; Cheong and Choi, 2012; Döring and Zernecke, 2012; Takeda et al., 2012; Van Brussel et al., 2013, 2014), experimental studies that already utilized DCs for immunotherapy of atherosclerosis are still quite limited (Habets et al., 2010; Hjerpe et al., 2010; van Es et al., 2010; Hermansson et al., 2011; Pierides et al., 2013). Currently, an immunotherapeutic strategy based on the isolation of autologous DCs, subsequent loading with appropriate antigen(s) *ex vivo* (e.g., immunogenic epitopes of modified LDL or a total plaque extract), and return to the host blood is under development (Habets et al., 2010; Hjerpe et al., 2010; van Es et al., 2010; Hermansson et al., 2011; Pierides et al., 2013) (Table 1). The future of this strategy is consisted in the development of atheroprotective vaccines on the basis of patient's DCs (Van Brussel et al., 2013).

**Table 1:** **Examples of anti-atherosclerosis immunization of experimental atherosclerosis animal models involving LDL or its related peptides**.

**Animals**	**Immunogenes**	**Effect on atherosclerosis**	**References**
LDLR-null rabbits	MDA-LDL	1.5-fold decrease in the extent of aortic lesions	Palinski et al., 1995
NZW rabbits on fat-rich diet	Native LDL or Cu^2+^-oxidized LDL	Reduction in aortic plaques by 74% (native LDL) and 48% (oxLDL)	Ameli et al., 1996
LDLR-null mice	Native LDL or MDA-LDL	Reduction in aortic sinus plaques by 46.3% (MDA-LDL) and 36.9% (native LDL)	Freigang et al., 1998
ApoE-null mice	MDA-LDL	2.1-fold decrease in the size of aortic sinus lesions	George et al., 1998
ApoE-null mice	apoB-100 peptides (p143 and p210)	Reduction in aortic atherosclerosis by 60%	Fredrikson et al., 2003
ApoE-null mice	Native LDL	1.7-fold decrease in the size of aortic sinus lesions	Chyu et al., 2004
ApoE-null mice	apoB-100 peptides (p143 and p210)	Reduction of aortic atherosclerosis by 40% and plaque inflammation by 89% (p210)	Chyu et al., 2005
LDLR-null mice	oxLDL or MDA-LDL	Attenuation of the initiation (30–71%) and progression (45%) of atherogenesis	van Puijvelde et al., 2006
LDLR-null apoB-transgenic mice	apoB-100 peptides (p45 and p210)	Reduction of aortic atherosclerosis by 66% (p45) and 59% (p210)	Klingenberg et al., 2010
ApoE-null mice	apoB peptide (p210)	Reduction in aortic sinus lesion size by 35%	Fredrikson et al., 2008
ApoE-null mice	DCs pulsed with MDA-LDL	Significant increase in aortic lesion size and inflammation	Hjerpe et al., 2010
LDLR-null mice	DCs pulsed with Cu^2+^-oxidized LDL	87% decrease in carotid artery lesion size and increase in plaque stability	Habets et al., 2010
LDLR-null mice	DCs transfected with FoxP3 mRNA	Reduction of Foxp3^+^ Tregs cells in several organs; increase in initial atherosclerotic lesion formation and in plaque cellularity	van Es et al., 2010
LDLR-null apoB100-transgenic mice	DCs loaded with apoB-100	70% reduction in aortic lesions and inflammation	Hermansson et al., 2011
ApoE-null mice	DCs loaded with apoB peptides (p2, p45, and p210)	50% decrease in plaque development	Pierides et al., 2013

apo, apolipoprotein; FoxP3, forkhead box P3; LDL, low density lipoprotein; LDLR, LDL receptor; MDA-LDL, malonaldehyde-modified LDL; NZW, New Zealand White; oxLDL, oxidized LDL; Tregs, regulatory T cells.

Another promising strategy involves studying properties of various immune cells interacting with DCs in order to develop DCs-based vaccines capable to modulate those immune cells in atherosclerosis. For example, van Es et al. (2010) used DCs expressing a FoxP3 transgene, a master regulator in the development and function of Tregs, to induce the anti-FoxP3 immune response in LDLR-null mice. The anti-FoxP3-specific immunity resulted in partial depletion of FoxP3-positive Tregs in several organs, early induction of proatherogenic inflammation and advanced atherosclerotic plaque progression. This observation indeed suggests for the crucial atheroprotective properties of Tregs.

An adoptive transfer of natural killer T (NKT) cells from Vα14Jα18 T-cell receptor transgenic mice caused significant progression in aortic atherosclerosis in recipient immune-deficient RAG-null LDLR-null mice (VanderLaan et al., 2007). Serum derived from the recipient animals then induced activation of Vα14Jα18 T-cell receptor-expressing hybridoma by DCs. This finding shows proatherogenic properties of CD1d-dependent Vα14 subpopulation of NKT cells that were likely to be stimulated by circulating endogenic lipoproteins in the atherosclerosis-prone animals (Rogers et al., 2008). Thus, such an approach may offer a tool for dissecting the contributions of individual subpopulations of particular immune cells to the process of atherogenesis. In the future, developing immune vaccines on the basis of specific subsets of DCs should be helpful for selective depletion of deleterious proatherogenic populations of effector cells such as Th1 and Th17 cells and induction of immunosuppressive anti-inflammatory Tregs (Van Brussel et al., 2013).

## Concluding remarks

It is appreciated nowadays that atherosclerosis is a chronic destruction of aortic and arterial walls, with a marked involvement of inflammation (Hansson and Hermansson, 2011; Tuttolomondo et al., 2012). DCs are recognized as key players in the induction of inflammatory response in the atherosclerotic plaque. It has become known that T cell activation occurs after a cell-to-cell-contact between a DC and a naïve T cells and by stimulation of the proinflammatory cytokines secreted by mature DCs. A proinflammatory function of mature DCs may be suppressed by a special subclass of T cells, namely by Tregs. On the other hand, immature DCs possess tolerogenic anti-inflammatory properties by leading naïve T cells to anergy or apoptosis and by inducing Tregs. Thus, depending on the maturation stage, DCs play a double role in atherogenesis: mature DCs display proatherogenic features whereas immature DCs seem to be anti-atherogenic. Since immature DCs are capable to induce Tregs and inhibit the inflammatory reaction in the atherosclerotic lesion, the development of strategies for the induction of tolerogenic DCs may be of great therapeutic promise.

### Conflict of interest statement

The authors declare that the research was conducted in the absence of any commercial or financial relationships that could be construed as a potential conflict of interest.

## Abbreviations

APCs: antigen-presenting cells
apo (Apo): apolipoprotein
*C. pneumonia*: *Chlamydia pneumonia*
CCL: CC-motif chemokines
CCR: chemokine receptors
CD: cluster of differentiation
cDCs: myeloid DCs
CTLA-4: cytotoxic T-lymphocyte antigen-4
DC: Dendritic cell
DCs: Dendritic cells
DNA: deoxyribonucleic acid
FoxP3: forkhead box P3
IDO: indoleamine 2,3-dioxigenase
IL: interleukin
iTregs: FoxP3+ Tregs
LDL: low density lipoprotein
LDLR: LDL receptor
LFA-1: lymphocyte function-associated antigen 1
M-CSF: macrophage-colony stimulating factor
MDA-LDL: malonaldehyde-modified LDL
MHC: major histocompatibility complex
NKT cells: natural killer T cells
NZW: New Zealand White
oxLDL: oxidized LDL
pDCs: plasmacytoid DCs
PDL-1: programmed death ligand 1
Scid: severe combined immunodeficiency syndrome
TGFßRII: tumor factor growth-ß receptor II
Th: T helper
Tr1: type 1 regulatory T cells
Tregs: regulatory T cells
VSMCs: vascular smooth muscle cells.
